# Depression, diabetes, comorbid depression and diabetes and risk of all-cause and cause-specific mortality: a prospective cohort study

**DOI:** 10.1007/s00125-022-05723-4

**Published:** 2022-05-27

**Authors:** Regina Prigge, Sarah H. Wild, Caroline A. Jackson

**Affiliations:** Usher Institute, Centre for Population Health Sciences, College of Medicine and Veterinary Medicine, University of Edinburgh, Edinburgh, UK

**Keywords:** Comorbidity, Complications, Depression, Diabetes, Interaction, Mortality, UK Biobank

## Abstract

**Aims/hypothesis:**

The aim of this study was to investigate the risks of all-cause and cause-specific mortality among participants with neither, one or both of diabetes and depression in a large prospective cohort study in the UK.

**Methods:**

Our study population included 499,830 UK Biobank participants without schizophrenia and bipolar disorder at baseline. Type 1 and type 2 diabetes and depression were identified using self-reported diagnoses, prescribed medication and hospital records. Mortality was identified from death records using the primary cause of death to define cause-specific mortality. We performed Cox proportional hazards models to estimate the risk of all-cause mortality and mortality from cancer, circulatory disease and causes of death other than circulatory disease or cancer among participants with either depression (*n*=41,791) or diabetes (*n*=22,677) alone and with comorbid diabetes and depression (*n*=3597) compared with the group with neither condition (*n*=431,765), adjusting for sociodemographic and lifestyle factors, comorbidities and history of CVD or cancer. We also investigated the interaction between diabetes and depression.

**Results:**

During a median of 6.8 (IQR 6.1–7.5) years of follow-up, there were 13,724 deaths (cancer, *n*=7976; circulatory disease, *n*=2827; other causes, *n*=2921). Adjusted HRs of all-cause mortality and mortality from cancer, circulatory disease and other causes were highest among people with comorbid depression and diabetes (HRs 2.16 [95% CI 1.94, 2.42]; 1.62 [95% CI 1.35, 1.93]; 2.22 [95% CI 1.80, 2.73]; and 3.60 [95% CI 2.93, 4.42], respectively). The risks of all-cause, cancer and other mortality among those with comorbid depression and diabetes exceeded the sum of the risks due to diabetes and depression alone.

**Conclusions/interpretation:**

We confirmed that depression and diabetes individually are associated with an increased mortality risk and also identified that comorbid depression and diabetes have synergistic effects on the risk of all-cause mortality that are largely driven by deaths from cancer and causes other than circulatory disease and cancer.

**Graphical abstract:**

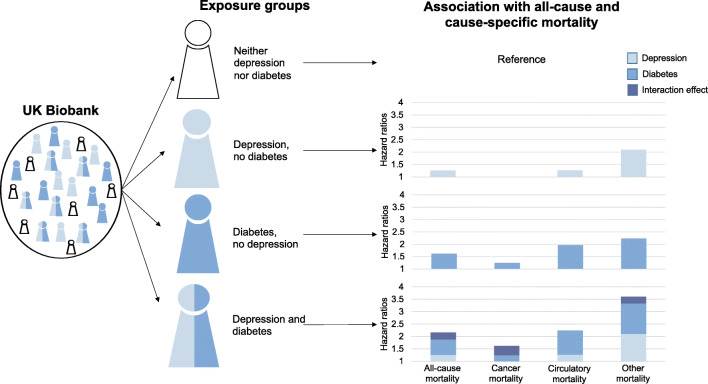

**Supplementary Information:**

The online version contains peer-reviewed but unedited supplementary material available at 10.1007/s00125-022-05723-4.

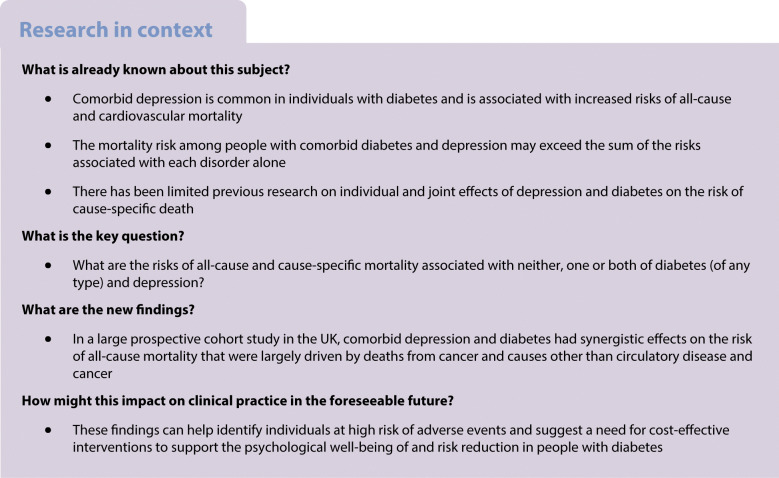



## Introduction

Depression is common among individuals with diabetes, with about 34% of women and 23% of men with type 2 diabetes having comorbid depression [1]. As such, individuals with diabetes are disproportionally affected by depression compared with the general population [1–3]. Importantly, those affected by both conditions are at higher risk of poor glycaemic control [4], medical non-compliance [5, 6] and micro- and macrovascular complications [7] than those with diabetes alone.

Previous meta-analyses have shown that comorbid depression is associated with an approximately 50–75% increased risk of all-cause mortality and 50% increased risk of cardiovascular mortality in individuals with type 1 or type 2 diabetes [8–12]. Importantly, as all of the studies included in these meta-analyses were based on patients with diabetes, the authors were not able to assess whether the presence of diabetes modified the excess mortality risk associated with depression.

There is some evidence that the association between depression and risk of all-cause mortality may be more pronounced among individuals with type 1 or type 2 diabetes than among those without [13–15], but few studies have investigated the individual and joint effects of depression and diabetes on mortality risk [13, 16–21]. These studies have consistently reported that people with comorbid depression and diabetes are at particularly high risk of all-cause and cardiac mortality. Furthermore, they have largely suggested that there are synergistic effects (supra-additive interaction) between depression and diabetes on risk of all-cause mortality and, to a lesser extent, on risk of cardiac mortality. None of the existing studies in the general population has investigated causes of death other than circulatory disease, despite evidence that depression may be linked to an increased risk of non-cardiovascular, non-cancer mortality in patients with diabetes [22]. Furthermore, existing studies are largely based on samples from the USA [16, 18–21], are limited by small sample sizes [16–18] or are based on selected patient populations, such as patients hospitalised for myocardial infarction [17, 20].

Despite the substantial burden of both depression and diabetes, and the potential impact on the prognosis of patients affected by both disorders, there is limited knowledge about the individual and joint effects of depression and diabetes on risk of death from specific causes. Therefore, the aim of this study was to investigate the risks of all-cause and cause-specific mortality among participants with neither, one or both of diabetes and depression in a large prospective cohort in the UK.

## Methods

### Study population

We included participants from the UK Biobank, a prospective cohort study of approximately 500,000 participants aged 40–69 years at baseline, recruited in 22 assessment centres in England, Scotland and Wales from 2006 to 2010 and followed up through linkage to routinely available national datasets [23]. Cohort entry date forms the baseline for this study. We excluded participants who withdrew from the study and whose information could not be linked to hospital or death records. Furthermore, those with a history of bipolar disorder or schizophrenia at baseline were excluded to avoid confounding by these additional severe mental illnesses. Analyses of UK Biobank data are conducted under generic approval from the NHS National Research Ethics Service (ref. 11/NW/0382, approval letter dated 17 June 2011). Full written informed consent was obtained from participants at the point of data collection.

### Exposure: depression, diabetes and their comorbidity

We defined depression as at least one of self-reported antidepressant use, self-reported doctor diagnosis of depression or hospital record of depression at baseline. Antidepressant use and self-reported doctor diagnosis of depression were identified in the nurse interview at baseline. In line with previous algorithms, we defined antidepressant use as self-reported use of at least one selective serotonin reuptake inhibitor or other antidepressant medication [24]. We defined hospital record of depression as diagnosis with a depressive episode or recurrent depressive episodes in hospital records (ICD-10: F32.X, F33.X) prior to recruitment to the study. We defined diabetes as self-reported diagnosis with, and/or treatment for, type 1 or 2 diabetes, or hospital record of type 1 or 2 diabetes at baseline (ICD-10: E10.X–E14.X). Self-reported diabetes was ascertained in the touchscreen questionnaire and nurse interview. Glucose-lowering treatment was defined as self-reported use of any medication listed in ‘A10 Drugs used in diabetes’ of the ATC/DDD index 2018 [25]. We used information on depression and diabetes at baseline to create our exposure variable, which consisted of four levels: neither depression nor diabetes, depression alone, diabetes alone, and both depression and diabetes.

### Outcome: all-cause and cause-specific mortality

We ascertained mortality from linked death records using the primary or underlying cause of death to define cause-specific mortality. Causes and dates of death were provided by NHS Digital for participants from England and Wales, and by the Information and Statistics Division for participants from Scotland [26]. We defined circulatory deaths using ICD-10 codes I00–I99 and G.45, cancer deaths using ICD-10 codes C00–C97, and other mortality as deaths from any other cause. We calculated survival times from the date of attending the baseline assessment centre to the date of death or the end of follow-up (31 November 2015).

### Covariates

Information on sociodemographic and lifestyle factors, comorbidities and family history was obtained through a self-report touchscreen questionnaire and nurse interviews and, where available, from values measured at the baseline assessment centre (see electronic supplementary material [ESM] Methods). Covariates included age, sex, ethnicity, income, education, area-based deprivation, BMI, smoking status, alcohol intake, physical activity level, fruit and vegetable intake, hypertension, high cholesterol level, history of CVD, history of cancer, family history of CVD and family history of severe depression.

### Statistical analyses

We performed analyses using R version 3.6.2 [27]. We assessed baseline characteristics across the four exposure groups. Because of the large sample size, we did not perform formal tests for differences between groups. We used Cox proportional hazards models to estimate HRs and 95% CIs for the risks of all-cause and cause-specific mortality among participants with either diabetes or depression alone and with comorbid diabetes and depression relative to the group with neither condition. The first model describes the unadjusted association; the second is adjusted for age, sex, ethnicity and socioeconomic factors (education, income and area-based deprivation); and the third additionally controls for BMI, alcohol intake, physical activity level, smoking status, fruit and vegetable consumption, oily fish intake, family history of CVD and depression, hypertension, high cholesterol level and history of CVD and cancer at baseline. We performed prespecified sex-stratified analyses to evaluate differences between men and women. As age did not fulfil the assumption of a linear contribution to the Cox proportional hazards model, we split the age distribution into four equally sized groups and included age as a categorical variable in the models. We tested the proportional hazards assumption for all variables using the cox.zph function, and investigated potential violations using log-minus-log survival plots and plots of scaled Schoenfeld residuals against time. We allowed for different baseline hazards for covariates that did not meet the proportional hazards assumption. Nonetheless, there was evidence of a violation of the proportional hazards assumption because of the small numbers of events towards the end of follow-up in one analysis. As truncation of follow-up at 6 years gave similar point estimates (ESM Table 1), the results for the whole follow-up are presented.

We tested for multiplicative interaction by adding a product term of depression and diabetes to the fully adjusted Cox proportional hazards model (and considered a two-sided *p*<0.05 statistically significant). We also investigated for additive (i.e. biological) interaction, which is more important for understanding population health, by calculating the relative excess risk due to interaction, the attributable proportion due to interaction and the synergy index, with accompanying 95% CIs [28].

Differences between participants with and without complete data were indicative of a violation of the missing completely at random assumption (ESM Table 2). For example, the group with complete information available included a higher proportion of men and a lower proportion of people with low socioeconomic status than the group with information missing for at least one variable. While the average number of variables with missing information among participants was low, overall missingness was high because of the large number of variables included, with 155,761 (31.2%) participants having at least one missing value for any variable. As the likely mechanism for missing information was deemed to be a missing at random mechanism, multiple imputation of missing data was performed using the mice package in R [29]. In keeping with the recommendation that the number of imputations should equate to the percentage of incomplete cases [30], we performed 32 imputations with 10 iterations. The imputations were run separately for participants with and without depression to take account of our interest in the interaction between depression and diabetes. As a complete case analysis is likely to be biased when missing data are not missing completely at random, the primary analyses are based on imputed data, with the results of the complete case analysis provided in ESM Tables 3–6.

### Sensitivity analysis

To facilitate comparisons with previous studies, we also performed a sensitivity analysis with subgroups of circulatory mortality as the outcome, specifically CVD mortality and non-CVD circulatory mortality. CVD mortality was defined as death from ischaemic heart disease, cerebrovascular disease or transient ischaemic attack (ICD-10: I20.X–I25.X, I60.X–I69.X, G45).

## Results

### Descriptive statistics

**Fig. 1:**
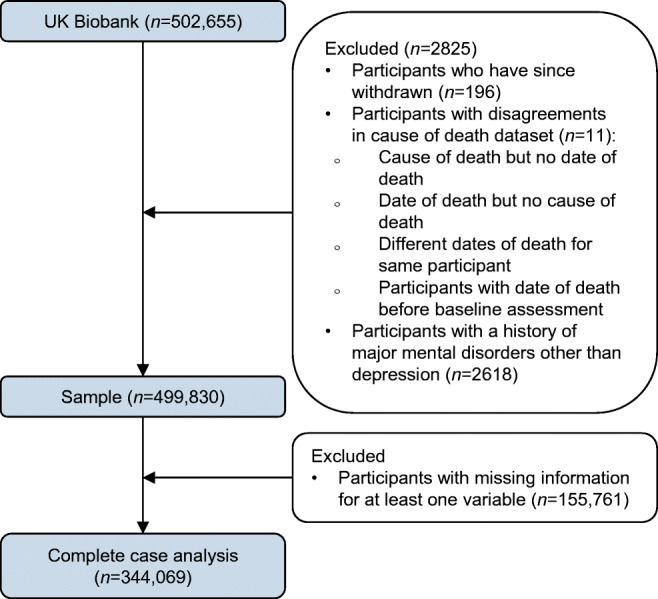
Flow diagram of sample selection

After excluding people with schizophrenia and bipolar disorder, our study population included 499,830 people (Fig. 1), with a median age of 58 (IQR 50–63) years at cohort entry, of whom 227,794 (45.6%) were male and 470,282 (94.1%) had a white ethnic background. At baseline, 431,765 (86.4%) participants had neither depression nor diabetes, 41,791 (8.4%) had depression alone, 22,677 (4.5%) had diabetes alone and 3597 (0.7%) had both diabetes and depression.

Baseline characteristics differed between exposure groups (Table 1). The group with diabetes alone was older and included a higher proportion of men than the other groups. Compared with the other groups, the group with comorbid depression and diabetes had the highest proportion with a low socioeconomic status and a higher prevalence of cardiovascular risk factors in general.

**Table 1:** Baseline characteristics and causes of death for UK Biobank participants with neither, one or both of depression and diabetes

Characteristic	Neither depression nor diabetes (*N*=431,765)	Depression alone (*N*=41,791)	Diabetes alone (*N*=22,677)	Depression and diabetes (*N*=3597)
Men	198,326 (45.9)	12,994 (31.1)	14,621 (64.5)	1853 (51.5)
Age (years), median (IQR)	58.0 (50.0, 63.0)	57.0 (50.0, 62.0)	61.0 (56.0, 66.0)	60.0 (54.0, 64.0)
Ethnicity				
White	407,309 (94.3)	40,224 (96.3)	19,451 (85.8)	3298 (91.7)
Other	22,165 (5.1)	1366 (3.3)	3009 (13.3)	270 (7.5)
Missing value	2291 (0.5)	201 (0.5)	217 (1.0)	29 (0.8)
Income (£)				
>100,000	21,400 (5.0)	959 (2.3)	500 (2.2)	26 (0.7)
52,000–100,000	78,918 (18.3)	4624 (11.1)	2328 (10.3)	205 (5.7)
31,000–51,999	98,415 (22.8)	7831 (18.7)	3798 (16.7)	430 (12.0)
18,000–30,999	92,559 (21.4)	9215 (22.1)	5229 (23.1)	740 (20.6)
<18,000	75,498 (17.5)	12,508 (29.9)	6521 (28.8)	1509 (42.0)
Missing value	64,975 (15.0)	6654 (15.9)	4301 (19.0)	687 (19.1)
Highest educational attainment				
College or university degree	142,744 (33.1)	11,419 (27.3)	5393 (23.8)	688 (19.1)
Other degree^a^	211,799 (49.1)	20,746 (49.6)	10,550 (46.5)	1649 (45.8)
None of the above	68,730 (15.9)	8865 (21.2)	6030 (26.6)	1149 (31.9)
Missing value	8492 (2.0)	761 (1.8)	704 (3.1)	111 (3.1)
Area-based deprivation				
1=least deprived	89,456 (20.7)	7116 (17.0)	3406 (15.0)	417 (11.6)
2	88,090 (20.4)	7451 (17.8)	3756 (16.6)	500 (13.9)
3	87,425 (20.2)	7907 (18.9)	4143 (18.3)	530 (14.7)
4	85,729 (19.9)	8558 (20.5)	4754 (21.0)	800 (22.2)
5=most deprived	80,556 (18.7)	10,686 (25.6)	6586 (29.0)	1346 (37.4)
Missing value	509 (0.1)	73 (0.2)	32 (0.1)	4 (0.1)
BMI				
Underweight or normal weight	149,232 (34.6)	12,062 (28.9)	2715 (12.0)	315 (8.8)
Overweight	185,461 (43.0)	16,727 (40.0)	7992 (35.2)	968 (26.9)
Obese	70,490 (16.3)	8481 (20.3)	6883 (30.4)	1085 (30.2)
Severely obese	18,093 (4.2)	2909 (7.0)	3086 (13.6)	671 (18.7)
Morbidly obese	6073 (1.4)	1315 (3.1)	1720 (7.6)	484 (13.5)
Missing value	2416 (0.6)	297 (0.7)	281 (1.2)	74 (2.1)
Physical activity				
High	155,724 (36.1)	12,098 (28.9)	6393 (28.2)	714 (19.8)
Moderate	168,937 (39.1)	15,666 (37.5)	8524 (37.6)	1222 (34.0)
Low	88,519 (20.5)	11,866 (28.4)	6451 (28.4)	1415 (39.3)
Missing value	18,585 (4.3)	2161 (5.2)	1309 (5.8)	246 (6.8)
Alcohol intake				
Risky drinking	177,718 (41.2)	13,362 (32.0)	7377 (32.5)	818 (22.7)
Safe drinking	188,146 (43.6)	19,939 (47.7)	10,736 (47.3)	1854 (51.5)
Missing value	65,901 (15.3)	8490 (20.3)	4564 (20.1)	925 (25.7)
Smoking status				
Never	240,327 (55.7)	20,265 (48.5)	10,385 (45.8)	1439 (40.0)
Previous	146,456 (33.9)	14,525 (34.8)	9687 (42.7)	1564 (43.5)
Current	42,601 (9.9)	6762 (16.2)	2342 (10.3)	561 (15.6)
Missing value	2381 (0.6)	239 (0.6)	263 (1.2)	33 (0.9)
Fruit and vegetable intake per day				
Less than five a day	300,939 (69.7)	28,969 (69.3)	14,610 (64.4)	2323 (64.6)
At least five a day	129,476 (30.0)	12,682 (30.3)	7914 (34.9)	1253 (34.8)
Missing value	1350 (0.3)	140 (0.3)	153 (0.7)	21 (0.6)
Oily fish intake				
At least once a week	241,217 (55.9)	21,512 (51.5)	12,728 (56.1)	1851 (51.5)
Less than once a week	142,095 (32.9)	13,991 (33.5)	6848 (30.2)	1128 (31.4)
Never	45,160 (10.5)	5924 (14.2)	2790 (12.3)	565 (15.7)
Missing value	3293 (0.8)	364 (0.9)	311 (1.4)	53 (1.5)
History of CVD	25,627 (5.9)	3901 (9.3)	4930 (21.7)	1079 (30.0)
History of cancer	37,186 (8.6)	4499 (10.8)	2226 (9.8)	415 (11.5)
Hypertension	236,516 (54.8)	23,461 (56.1)	19,188 (84.6)	3088 (85.8)
High cholesterol level	66,241 (15.3)	8529 (20.4)	17,734 (78.2)	2966 (82.5)
Family history of CVD	297,673 (68.9)	29,933 (71.6)	15,749 (69.4)	2605 (72.4)
Family history of depression	34,480 (8.0)	7646 (18.3)	1425 (6.3)	530 (14.7)
Cause of death				
All-cause	10,429 (2.4)	1453 (3.5)	1492 (6.6)	350 (9.7)
Of which				
Cancer^b^	6520 (62.5)	708 (48.7)	613 (41.1)	135 (38.6)
Circulatory disease^b^	1947 (18.7)	277 (19.1)	499 (33.4)	104 (29.7)
Other causes^b^	1962 (18.8)	468 (32.2)	380 (25.5)	111 (31.7)

Data are *n* (%) unless otherwise indicated

^a^A level, O level, Certificate of Secondary Education, National Vocational Qualification or equivalent

^b^The denominators for calculating the percentage values are the numbers who have died in each group

During a median of 6.8 (IQR 6.1–7.5) years of follow-up, there were 13,724 deaths, 7976 of which were from cancer and 2827 and 2921 of which were from circulatory disease and other causes, respectively (Table 1). For all causes of death, the proportions of participants who died were lowest in the group with neither depression nor diabetes followed by the groups with depression alone and diabetes alone and highest among those with both depression and diabetes. Overall, the three most frequent other causes of death were respiratory diseases, diseases of the digestive system and external causes of mortality. A detailed description of other causes of death by exposure group is presented in ESM Table 7.

### Associations with all-cause and cause-specific mortality

Diabetes alone and comorbid diabetes and depression were associated with greater risks of all-cause and cause-specific mortality relative to participants with neither depression nor diabetes in all models (Table 2). Depression alone was also associated with greater risks of all-cause and circulatory mortality and mortality from other causes, but not cancer mortality. The associations were attenuated but remained statistically significant after adjusting for various factors. In the fully adjusted model, compared with those with neither condition, the risk of all-cause mortality was 26% higher among people with depression alone (HR 1.26, 95% CI 1.19, 1.33), 62% higher among individuals with diabetes alone (HR 1.62, 95% CI 1.52, 1.72) and 116% higher among people with comorbid diabetes and depression (HR 2.16, 95% CI 1.94, 2.42). The associations followed the same pattern in all models, with the largest effect sizes seen for those with both depression and diabetes and the lowest effect sizes seen for those with depression alone.

**Table 2:** HRs (95% CIs) for all-cause and cause-specific mortality risk among UK Biobank participants with neither, one or both of depression and diabetes

Causes of mortality	Exposure	Unadjusted HR (95% CI)	Adjusted HR (95% CI)	
			Model 1^a^	Model 2^b^
All-cause mortality	Neither depression nor diabetes	1.0	1.0	1.0
	Depression alone	1.44 (1.36, 1.52)	1.45 (1.37, 1.53)	1.26 (1.19, 1.33)
	Diabetes alone	2.82 (2.67, 2.97)	1.86 (1.76, 1.97)	1.62 (1.52, 1.72)
	Depression and diabetes	4.19 (3.77, 4.66)	2.88 (2.59, 3.21)	2.16 (1.94, 2.42)
Cancer mortality	Neither depression nor diabetes	1.0	1.0	1.0
	Depression alone	1.12 (1.04, 1.21)	1.12 (1.04, 1.22)	1.00 (0.92, 1.08)
	Diabetes alone	1.85 (1.70, 2.01)	1.31 (1.21, 1.43)	1.24 (1.13, 1.36)
	Depression and diabetes	2.58 (2.18, 3.06)	1.91 (1.61, 2.27)	1.62 (1.35, 1.93)
Circulatory mortality	Neither depression nor diabetes	1.0	1.0	1.0
	Depression alone	1.47 (1.30, 1.67)	1.56 (1.37, 1.77)	1.27 (1.12, 1.45)
	Diabetes alone	5.04 (4.57, 5.56)	2.89 (2.61, 3.20)	1.97 (1.76, 2.20)
	Depression and diabetes	6.66 (5.47, 8.11)	4.12 (3.37, 5.03)	2.22 (1.80, 2.73)
Mortality from other causes	Neither depression nor diabetes	1.0	1.0	1.0
	Depression alone	2.46 (2.23, 2.72)	2.38 (2.14, 2.63)	2.10 (1.89, 2.34)
	Diabetes alone	3.82 (3.43, 4.27)	2.41 (2.16, 2.70)	2.23 (1.97, 2.53)
	Depression and diabetes	7.07 (5.84, 8.57)	4.43 (3.65, 5.38)	3.60 (2.93, 4.42)

^a^Model 1: adjusted for age, sex, ethnicity, education, income and area-based deprivation

^b^Model 2: adjusted as in model 1 plus for BMI, physical activity level, alcohol intake, smoking status, fruit and vegetable intake, oily fish intake, high cholesterol level, hypertension, history of CVD, history of cancer, family history of CVD and family history of depression at baseline

The associations for the groups with depression alone and diabetes alone were mostly similar in men and women (Table 3). However, the HR of circulatory mortality among those with diabetes alone and the HR of other mortality among those with depression and diabetes were higher for women than men. However, the 95% CIs were wide and overlapped.

**Table 3:** HRs (95% CIs) for all-cause and cause-specific mortality risk among UK Biobank participants with neither, one or both of depression and diabetes stratified by sex

Causes of mortality	Exposure	Adjusted HR (95% CI)^a^	
		Men	Women
All-cause mortality	Neither depression nor diabetes	1.0	1.0
	Depression alone	1.30 (1.20, 1.41)	1.24 (1.14, 1.34)
	Diabetes alone	1.63 (1.52, 1.75)	1.59 (1.41, 1.80)
	Depression and diabetes	2.10 (1.83, 2.41)	2.28 (1.88, 2.76)
Cancer mortality	Neither depression nor diabetes	1.0	1.0
	Depression alone	0.96 (0.85, 1.09)	1.04 (0.94, 1.15)
	Diabetes alone	1.23 (1.11, 1.38)	1.26 (1.07, 1.49)
	Depression and diabetes	1.63 (1.30, 2.03)	1.58 (1.18, 2.12)
Circulatory mortality	Neither depression nor diabetes	1.0	1.0
	Depression alone	1.32 (1.12, 1.55)	1.21 (0.97, 1.51)
	Diabetes alone	1.91 (1.69, 2.16)	2.28 (1.77, 2.95)
	Depression and diabetes	2.22 (1.74, 2.82)	2.24 (1.45, 3.46)
Mortality from other causes	Neither depression nor diabetes	1.0	1.0
	Depression alone	2.11 (1.83, 2.44)	2.08 (1.78, 2.44)
	Diabetes alone	2.27 (1.97, 2.63)	2.19 (1.69, 2.83)
	Depression and diabetes	3.13 (2.40, 4.08)	4.51 (3.24, 6.27)

^a^Fully adjusted model: adjusted for age, ethnicity, education, income, area-based deprivation, BMI, physical activity level, alcohol intake, smoking status, fruit and vegetable intake, oily fish intake, high cholesterol level, hypertension, history of CVD, history of cancer, family history of CVD and family history of depression at baseline

### Additive and multiplicative interaction

Among the group with comorbid diabetes and depression, the risks of all-cause, cancer and other mortality exceeded the sum of the risks due to diabetes alone and depression alone (ESM Fig. 1), suggesting the presence of an interaction on the additive scale. However, formal statistical evidence of an additive interaction was found only for all-cause and cancer mortality (relative excess risk due to interaction 0.29 [95% CI 0.03, 0.54] and 0.38 [95% CI 0.07, 0.68], respectively) (ESM Table 8). Furthermore, there was evidence of an interaction between depression and diabetes on the multiplicative scale for cancer mortality (*p*=0.006) but not for all-cause mortality, circulatory mortality and mortality from other causes (*p*=0.182, 0.578 and 0.061, respectively).

### Sensitivity analyses

Depression alone, diabetes alone and comorbid diabetes and depression were associated with greater risks of both CVD mortality and non-CVD circulatory mortality relative to participants with neither depression nor diabetes in unadjusted and partially adjusted models. In the fully adjusted model, the association with risks of CVD mortality and non-CVD circulatory mortality was strongest among the group with comorbid depression and diabetes. While the association with risk of CVD mortality was weakest among those with depression alone, the association with non-CVD circulatory mortality risk was similar for those with depression alone and diabetes alone. For non-CVD circulatory mortality, the risk among the group with comorbid depression and diabetes exceeded the sum of the risks due to depression alone and diabetes alone (ESM Table 9). There was no evidence of multiplicative or additive interaction for either CVD mortality risk or non-CVD circulatory mortality risk (ESM Table 8).

## Discussion

### Principal findings

In a large prospective study in the UK, we confirmed the higher mortality risk associated with each of depression and diabetes, and also identified synergistic effects of depression and diabetes on different causes of mortality beyond that expected from their individual effects. This pattern remained even after adjusting for a wide range of potential confounding factors.

### Strengths and limitations of this study

Our final model adjusted for a number of covariates that could be potential mediators of the observed associations. An important consideration when distinguishing between confounders and mediators is whether factors change over time and do not or do lie on the causal pathway, respectively [30]. While genetically determined factors such as sex and ethnicity clearly preceded the onset of our exposure and outcome and may confound their association, lifestyle factors and physical measures such as smoking status and BMI could be potential mediators of the observed associations. By adjusting for these covariates we can identify the extent to which any association is independent of these factors. The pattern of results was similar in models before and after the inclusion of potential mediators (ESM Figs 1, 2). However, our final model may have underestimated the strength of the association between depression and/or diabetes and risk of mortality and the magnitude of the interaction effect between depression and diabetes on risk of circulatory diseases. Further research is required to perform a formal mediation analysis, ideally using a dataset with time-varying information on covariates, if criteria for establishing causality are met [31].

Our study has a number of strengths. It is one of just a few cohort studies investigating the relative importance of depression and diabetes and their synergistic effects on risk of all-cause and circulatory mortality in the general population and within a universal healthcare setting. Furthermore, to our knowledge, this is the first study to describe the effects of comorbid depression and diabetes on risk of mortality from cancer and causes of death other than circulatory disease and cancer. We used a large prospective cohort that contained detailed information on a range of potential confounding factors. The large sample size and subsequent large number of deaths meant we had sufficient power to investigate the individual and synergistic effects of our exposure groups, to study cause-specific mortality risks and to stratify our analysis by sex. A further advantage of our study is that, in contrast to many other prospective cohort studies, there was limited attrition, because we relied on administrative health records to ascertain outcomes.

Our study has some limitations. The UK Biobank had a low response rate (5.5%), which resulted in a relatively healthy cohort from a higher socioeconomic background than that of the general population [32]. However, it has been argued that this is unlikely to influence estimates of associations between diseases, given that there are large numbers of participants with different levels of risk factors in the sample [32]. Nonetheless, selection bias might have influenced some of the results of this analysis. As previously described, selection into a cohort can introduce collider bias that can work in any direction [33]. However, without further information on the population from which the cohort is drawn or from unselected cohorts, it is not possible to determine the presence of bias or the direction of bias in the strength of the association.

In addition, there is potential for misclassification because participants may have under-reported depression, diabetes and comorbid depression and diabetes at baseline. Although our measurement at baseline used hospital records and self-report, it is possible that we misclassified some participants’ exposure status and have underestimated the mortality risks associated with depression and diabetes. We may have further inaccurately estimated the mortality risks associated with the presence of diabetes, as we did not identify individuals with undiagnosed diabetes. Furthermore, individuals may have been misclassified as depressed if they took antidepressants for treatment of chronic pain, such as neuropathic pain. This could lead to overestimation of mortality risks associated with depression if chronic pain is associated with higher mortality risks than depression (and underestimation if chronic pain is associated with lower mortality risks). We were not able to update exposure status during follow-up. In addition, we may have missed a small number of deaths occurring outside the UK, but this is likely to have occurred non-differentially across the four exposure groups. This may, however, have further biased our findings towards the null. Although key confounding factors were adjusted for in this analysis, residual confounding might explain some of the observed effect, for example if the measurement error of lifestyle factors and comorbidities was systematically different among the four exposure groups.

### Strengths and weaknesses in relation to other studies

Our findings are in keeping with previous studies reporting a high risk of all-cause and circulatory mortality risk among people with comorbid depression and diabetes that exceeds the risk due to having either diabetes or depression alone [13, 16–21]. While the strengths of the associations between comorbid depression and diabetes and risk of all-cause and circulatory mortality were similar in some previous studies [17–19, 21], others observed much higher HRs of 3.64 [20], 3.71 [13] and 4.56 [16] for risk of all-cause mortality and 3.27 for circulatory mortality risk [17]. Potential explanations for the observed differences are the use of very selected reference groups, such as people with a score of 0 on the Centre for Epidemiologic Studies of Depression Scale [13, 16], and differences in the study populations [17, 20].

Our study uniquely extends these findings to risk of cancer mortality and causes of death other than circulatory disease and cancer. In patients with diabetes, a previous study reported an increased risk of non-CVD, non-cancer mortality in people with comorbid depression and diabetes, whereas there was no association with risk of CVD and cancer mortality [22]. However, with small number of deaths in some groups, this study may have been underpowered to detect statistically significant differences. As this study was based on patients with diabetes, the joint effect of depression and diabetes could not be examined.

### Possible explanations for our findings

The underlying mechanisms for the synergistic effect of depression and diabetes on mortality risk remain to be established. As we found synergistic effects of depression and diabetes for risk of different causes of mortality, it is unlikely that the underlying mechanism is organ or disease specific [34]. A more general explanation for the excess mortality risk among those affected by both depression and diabetes is that depression might make adoption and maintenance of a healthy lifestyle, including smoking cessation and self-management, more difficult. For example, depression has been shown to be a risk factor for medical non-compliance among individuals with comorbidities [5, 6], which might lead to adverse effects such as poor glycaemic control among individuals with comorbid depression and diabetes [4]. Second, individuals with mental–physical comorbidity may receive suboptimal quality of care, which in turn may increase their risk of adverse events [35, 36]. As such, the negative consequences of depression and diabetes may be aggravated among those with comorbid depression and diabetes because of the lack of successful treatment or self-management strategies for both conditions. However, more research is needed to further explore this hypothesis.

### Implications of this study and future research

Our findings highlight the scope for improved care and treatment of people with depression, particularly those with diabetes. Despite the availability of guidelines on encouraging psychological well-being in people with diabetes [37], depression continues to be overlooked in clinical practice [38]. Screening for depression in clinical practice, particularly among those with diabetes, may be a helpful first step to identify patients at high risk of adverse effects. However, a requirement of screening programmes is the provision of cost-effective interventions to individuals identified as being at high risk of adverse events. This is particularly challenging in this context due to the lack of cost-effective interventions to reduce adverse outcomes in this patient group [39–41]. An RCT found that allocating a trained depression care manager and offering an antidepressant and interpersonal psychotherapy to patients with comorbid depression and diabetes may reduce the 5 year mortality rate [42]. However, the statistical methods used by Bogner et al [42] were criticised as they may have resulted in model overfitting [43], and few health systems are likely to have the resources to provide such interventions to the large number of people who might be eligible. Thus, further RCTs are needed to identify cost-effective interventions that reduce the risk of mortality and improve quality of life in patients with one or both of depression and diabetes.

A particular focus of future studies should be the potential synergistic effect of depression and diabetes not only on risk of circulatory mortality but also on cancer mortality and mortality from other causes, as this is the first study to report this. It would be helpful to establish whether the synergistic effect of depression and diabetes on mortality risk is observed in other settings and for participants with type 1 and type 2 diabetes. Furthermore, future studies should attempt to identify mechanisms that may be responsible for the synergistic effect of depression and diabetes on risk of mortality in order to inform the development and testing of interventions. Finally, the temporality of depression and diabetes deserves further attention, with one recent study suggesting smaller joint effects of depression and diabetes when both disorders are ascertained at the same point in time than when depressive symptoms develop after diagnosis of diabetes [18].

### Conclusions

In summary, we found that individuals with depression and diabetes were at high risk of all-cause mortality and mortality from cancer, circulatory disease and causes other than circulatory disease or cancer. In the fully adjusted model, the combined association between depression and diabetes was additive for risk of circulatory mortality and synergistic (i.e. supra-additive) for risk of cancer and mortality from causes other than circulatory disease and cancer (described in Table 2, ESM Fig. 1). Although some progress has been made in the past, our findings highlight the need for further research and the potential for the improved treatment of depression, particularly in people with diabetes.

## Supplementary Information

ESM 1 (PDF 197 kb)

## Data Availability

All bona fide researchers in academic, commercial and charitable settings can apply to use the UK Biobank resource for health-related research in the public interest (www.ukbiobank.ac.uk/register-apply/).
