# Supplementary material for: Depression, diabetes, comorbid depression and diabetes and risk of all-cause and cause-specific mortality: a prospective cohort study
Source: Diabetologia. 2022 May 27;65(9):1450–60. doi: 10.1007/s00125-022-05723-4 (PMC9345808; doi:10.1007/s00125-022-05723-4)
Supplement: Supplementary file 1 — (PDF 197 kb) [file 125_2022_5723_MOESM1_ESM.pdf]

Electronic supplementary material

ESM methods: Ascertainment of covariates

The following covariates were ascertained through self-report in the touchscreen questionnaire at the baseline assessment. Income was defined as average household income before tax, with participants selecting one of five categories. Education was defined as highest educational attainment, which we categorized into: College or university degree; A level, O level, Certificate of Secondary Education or National Vocational Qualification, or equivalent; and none of the above). Area-based deprivation was assessed through the Townsend Deprivation Index (1), which we divided into fifths for this study. Due to small numbers of participants with a non-white ethnicity, we categorised ethnicity as white or other ethnicity. We categorised smoking as never, previous, or current smoker. We defined alcohol intake as safe drinking if men or women consumed ≤ 14 units of alcohol per week and as risky drinking if alcohol intake exceeded 14 units per week (2). In accordance with the International Physical Activity Questionnaire (3), we assigned level of physical activity as low, moderate or high. We classified oily fish intake as at least once a week, less than once per week, and never. We used information on daily fruit and vegetable intake to define consumption of at least five fruits or vegetables per day (4). Family history of stroke, heart disease, high blood pressure, and severe depression were defined as self-reported illness of mother or father. We calculated body mass index (kg/m^2, BMI) based on measured height and weight ascertained at the assessment centre at baseline, and categorised this as: under- or normal weight (<18.5 – 24.9 kg/m^2); overweight (25 – 29.9 kg/m^2); obese (30 – 34.9 kg/m^2); severely obese (35 – 39.9 kg/m^2); and morbidly obese (≥40 kg/m^2). Since there were so few participants underweight (0.5% of all participants included in the analysis), these were included in the same category as normal weight. We defined

hypertension and high cholesterol level as diagnosis and/or treatment, ascertained through self-report in the touchscreen questionnaire or nurse interview. Additionally, we identified participants with hypertension through blood pressure measurements of ≥140/90 mmHg at the assessment centre at baseline. We ascertained history of CVD (stroke, myocardial infarction, angina or transient ischaemic attack) and history of cancer using responses to the touchscreen questionnaire and nurse interview, and from linked hospital admission records prior to baseline.

References

1. Townsend P: Deprivation. Journal of Social Policy 1987; 16:125-146
2. Department of Health (Ed.). UK Chief Medical Officers' Low Risk Drinking Guidelines 2016
3. Guidelines for Data Processing and Analysis of the International Physical Activity Questionnaire (IPAQ) - Short Form [article online], 2010. Available from https://sites.google.com/site/theipaq/scoring-protocol. Accessed 23/10/2017
4. World Health Organization (Ed.). Fruit and vegetables for health: report of the Joint FAO/WHO Workshop on Fruit and Vegetables for Health, 1-3 September 2004, Kobe, Japan. Geneva, 2005

ESM Table 1: HRs (95% CIs) for CVD mortality risk among UK Biobank participants with neither, one or both of depression and diabetes with truncation of follow-up at six years

**Cause of mortality**	**Exposure**	**Unadjusted HR (95%CI)**	**Adjusted HR (95%CI)**	
			Model 1[a]	Model 2[b]
**CVD mortality**	Neither depression nor diabetes	1.0	1.0	1.0
	Depression alone	1.41 (1.14 – 1.74)	1.51 (1.22 – 1.87)	1.24 (1.00 – 1.54)
	Diabetes alone	4.94 (4.19 – 5.83)	2.74 (2.31 – 3.24)	1.95 (1.62 – 2.34)
	Depression and diabetes	9.39 (6.97 – 12.64)	5.25 (3.88 – 7.09)	3.00 (2.19 – 4.11)

a Model 1: adjusted for age, sex, ethnicity, education, income and area-based deprivation
b Model 2: adjusted as in model 1 plus for BMI, physical activity level, alcohol intake, smoking status, fruit and vegetable intake, oily fish intake, high cholesterol level, hypertension, history of CVD, history of cancer, family history of CVD and family history of depression at baseline

ESM Table 2: Baseline characteristics and causes of death, separately for UK Biobank participants with and without complete data available

**Characteristic**	**Incomplete cases** (*N* = 155,761)	**Complete cases** (*N* = 344,069)
**Men**	57,446 (36.9)	170,348 (49.5)
**Age (years),** median (IQR)	59.0 (51.0, 64.0)	57.0 (50.0, 63.0)
**White ethnicity**	141,511 (90.9)	328771 (95.6)
**Income (£)**		
> 100,000	1766 (2.2)	21,119 (6.1)
52,000 – 100,000	10,284 (13.0)	75,791 (22.0)
31,000 – 51,999	18,622 (23.5)	91,852 (26.7)
18,000 – 30,999	22,108 (27.9)	85,635 (24.9)
< 18,000	26,364 (33.3)	69,672 (20.2)
**Highest educational attainment**		
College or university degree	32,666 (22.4)	127,578 (37.1)
Other degrees[a]	74,374 (51.0)	170,370 (49.5)
None of the above	38,653 (26.5)	46,121 (13.4)
**Area-based deprivation**		
1 = least deprived	28,679 (18.5)	71,716 (20.8)
2	28,929 (18.6)	70,868 (20.6)
3	29,983 (19.3)	70,022 (20.4)
4	30,356 (19.6)	69,485 (20.2)
5 = most deprived	37,196 (24.0)	61,978 (18.0)
**BMI**		
Underweight or normal weight	47,239 (30.9)	117,085 (34.0)
Overweight	61,893 (40.5)	149,255 (43.4)
Obese	29,546 (19.3)	57,393 (16.7)
Severely obese	9730 (6.4)	15,029 (4.4)
Morbidly obese	4285 (2.8)	5307 (1.5)
**Physical activity**		
High	45,348 (34.0)	129,581 (37.7)
Moderate	52,871 (39.6)	141,478 (41.1)
Low	35,241 (26.4)	73,010 (21.2)
**Alcohol intake** = Risky drinking	29,266 (38.6)	170,009 (49.4)
**Smoking status**		
Never	87,498 (57.2)	184,918 (53.7)
Previous	47,500 (31.1)	124,732 (36.3)
Current	17,847 (11.7)	34,419 (10.0)
**Fruit and vegetable intake per day** = Less than five a day	104,873 (68.1)	241,968 (70.3)
**Oily fish intake**		
At least once a week	82,387 (54.3)	194,921 (56.7)
Less than once a week	49,238 (32.4)	114,824 (33.4)
Never	20,115 (13.3)	34,324 (10.0)
**History of CVD**	13,067 (8.4)	22,470 (6.5)
**History of cancer**	14,498 (9.3)	29,828 (8.7)
**Hypertension**	91,238 (58.6)	191,015 (55.5)
**High cholesterol level**	32,544 (20.9)	62,926 (18.3)
**Family history of CVD**	106,112 (68.1)	239,848 (69.7)
**Family history of depression**	12,656 (8.1)	31,425 (9.1)
**Cause of death**		
Cancer	2872 (1.8)	5104 (1.5)
Circulatory disease	1079 (0.7)	1748 (0.5)
Other causes	1212 (0.8)	1709 (0.5)

Data are number (%) unless otherwise indicated.

a A level, O level, Certificate of secondary education, National vocational qualification, or equivalent

ESM Table 3: HRs (95% CIs) for all-cause and cause-specific mortality risk among UK Biobank participants with neither, one of both of depression and diabetes (complete case analysis)

Causes of mortality	Exposure	Unadjusted HR (95% CI)	Adjusted HR (95% CI)	
			Model 1[a]	Model 2[b]
**All-cause mortality**	Neither depression nor diabetes	1.0	1.0	1.0
	Depression alone	1.36 (1.26 – 1.46)	1.37 (1.27 – 1.47)	1.19 (1.11 – 1.29)
	Diabetes alone	2.81 (2.61 – 3.02)	1.79 (1.67 – 1.93)	1.56 (1.45 – 1.69)
	Depression and diabetes	4.69 (4.07 – 5.41)	3.02 (2.62 – 3.48)	2.32 (2.00 – 2.69)
**Cancer mortality**	Neither depression nor diabetes	1.0	1.0	1.0
	Depression alone	1.05 (0.94 – 1.16)	1.05 (0.94 – 1.16)	0.93 (0.83 – 1.03)
	Diabetes alone	2.00 (1.80 – 2.22)	1.36 (1.23 – 1.52)	1.28 (1.14 – 1.43)
	Depression and diabetes	2.82 (2.24 – 3.54)	1.96 (1.56 – 2.47)	1.70 (1.35 – 2.15)
**Circulatory mortality**	Neither depression nor diabetes	1.0	1.0	1.0
	Depression alone	1.37 (1.16 – 1.62)	1.47 (1.24 – 1.74)	1.22 (1.02 – 1.44)
	Diabetes alone	4.71 (4.13 – 5.38)	2.61 (2.28 – 2.99)	1.82 (1.57 – 2.10)
	Depression and diabetes	7.55 (5.83 – 9.79)	4.31 (3.32 – 5.59)	2.41 (1.84 – 3.16)
**Mortality from other causes**	Neither depression nor diabetes	1.0	1.0	1.0
	Depression alone	2.47 (2.16 – 2.82)	2.37 (2.07 – 2.72)	2.11 (1.84 – 2.43)
	Diabetes alone	3.68 (3.16 – 4.28)	2.24 (1.92 – 2.61)	2.10 (1.77 – 2.48)
	Depression and diabetes	8.36 (6.47 – 10.79)	4.83 (3.73 – 6.25)	3.85 (2.93 – 5.06)

a Model 1: adjusted for age, sex, ethnicity, education, income and area-based deprivation
b Model 2: adjusted as in model 1 plus for BMI, physical activity level, alcohol intake, smoking status, fruit and vegetable intake, oily fish intake, high cholesterol level, hypertension, history of CVD, history of cancer, family history of CVD and family history of depression at baseline

ESM Table 4: HRs (95% CIs) for all-cause and cause-specific mortality risk among UK Biobank participants with neither, one or both of depression and diabetes stratified by sex (complete case analysis)

Causes of mortality	Exposure	Adjusted HR (95% CI)[a]	
		Men	Women
**All-cause mortality**	Neither depression nor diabetes	1.0	1.0
	Depression alone	1.24 (1.12 – 1.38)	1.15 (1.03 – 1.29)
	Diabetes alone	1.58 (1.44 – 1.72)	1.53 (1.28 – 1.83)
	Depression and diabetes	2.40 (2.02 – 2.84)	2.09 (1.56 – 2.81)
**Cancer mortality**	Neither depression nor diabetes	1.0	1.0
	Depression alone	0.87 (0.73 – 1.02)	0.99 (0.86 – 1.14)
	Diabetes alone	1.28 (1.12 – 1.46)	1.27 (1.01 – 1.60)
	Depression and diabetes	1.86 (1.41 – 2.45)	1.38 (0.88 – 2.17)
**Circulatory mortality**	Neither depression nor diabetes	1.0	1.0
	Depression alone	1.30 (1.06 – 1.59)	1.03 (0.74 – 1.43)
	Diabetes alone	1.75 (1.50 – 2.06)	2.35 (1.62 – 3.40)
	Depression and diabetes	2.53 (1.88 – 3.40)	1.95 (0.97 – 3.92)
**Mortality from other causes**	Neither depression nor diabetes	1.0	1.0
	Depression alone	2.16 (1.81 – 2.58)	2.01 (1.61 – 2.52)
	Diabetes alone	2.18 (1.81 – 2.63)	1.84 (1.22 – 2.76)
	Depression and diabetes	3.49 (2.51 – 4.84)	4.76 (2.91 – 7.80)

a Fully adjusted model: adjusted for age, ethnicity, education, income and area-based deprivation, BMI, physical activity level, alcohol intake, smoking status, fruit and vegetable intake, oily fish intake, high cholesterol level, hypertension, history of CVD, history of cancer, family history of CVD and family history of depression at baseline

ESM Table 5: Measures of additive and multiplicative interaction between depression and diabetes on risk of all-cause and cause-specific mortality (complete case analysis)

Causes of mortality	Additive interaction			Multiplicative interaction
	RERI (CI)	***AP (CI)***	***S (CI)***	***p-value***
**All-cause mortality**	0.56 (0.21 – 0.92)	0.24 (0.12 – 0.36)	1.74 (1.28 – 2.37)	0.003
**Cancer mortality**	0.38 (-0.30 – 1.06)	0.16 (-0.09 – 0.40)	1.37 (0.81 – 2.31)	0.005
**Circulatory mortality**	0.50 (0.08 – 0.92)	1.37 (0.81 – 2.31)	3.47 (1.27 – 9.49)	0.294
**Mortality from other causes**	0.64 (-0.41 – 1.70)	0.17 (-0.07 – 0.40)	1.29 (0.88 – 1.90)	0.658
**CVD mortality**	0.45 (-0.38 – 1.28)	0.17 (-0.10 – 0.44)	1.38 (0.80 – 2.36)	0.321
**Non-CVD circulatory mortality**	0.17 (-0.99 – 1.32)	0.10 (-0.52 – 0.72)	1.30 (0.23 – 7.41)	0.697

AP: Attributable proportion, RERI: Relative excess risk for interaction, S: Synergy index

ESM Table 6: HRs (95% CIs) for CVD- and non-CVD circulatory mortality risk among UK Biobank participants with neither, one or both of depression and diabetes (complete case analysis)

**Causes of mortality**	**Exposure**	**Unadjusted HR (95%CI)**	**Adjusted HR (95%CI)**	
			Model 1[a]	Model 2[b]
**CVD mortality**	Neither depression nor diabetes	1.0	1.0	1.0
	Depression alone	1.37 (1.13 – 1.67)	1.47 (1.21 – 1.79)	1.20 (0.98 – 1.46)
	Diabetes alone	5.25 (4.53 – 6.08)	2.87 (2.47 – 3.34)	2.00 (1.70 – 2.36)
	Depression and diabetes	8.43 (6.32 –11.23)	4.69 (3.51 – 6.27)	2.65 (1.96 – 3.59)
**Non-CVD circulatory mortality**	Neither depression nor diabetes	1.0	1.0	1.0
	Depression alone	1.36 (0.98 – 1.88)	1.48 (1.07 – 2.06)	1.27 (0.91 – 1.78)
	Diabetes alone	3.24 (2.40 – 4.37)	1.87 (1.38 – 2.54)	1.27 (0.92 – 1.77)
	Depression and diabetes	5.17 (2.84 – 9.43)	3.16 (1.73 – 5.79)	1.71 (0.92 – 3.20)

a Model 1: adjusted for age, sex, ethnicity, education, income and area-based deprivation
b Model 2: adjusted as in model 1 plus for BMI, physical activity level, alcohol intake, smoking status, fruit and vegetable intake, oily fish intake, high cholesterol level, hypertension, history of CVD, history of cancer, family history of CVD and family history of depression at baseline

ESM Table 7: Causes of death in the other mortality group, overall and separately for participants with none, one or both of depression and diabetes (n, %)

**Causes of death**	**Neither depression nor diabetes** (*N* = 1,962)	**Depression alone** (*N* = 468)	**Diabetes alone** (*N* = 380)	**Depression and diabetes** (*N* = 111)	**Total** (*N* = 2,921)
Diseases of the respiratory system	517 (26.4%)	132 (28.2%)	101 (26.6%)	24 (21.6%)	774 (26.5%)
Diseases of the digestive system	343 (17.5%)	88 (18.8%)	76 (20.0%)	23 (20.7%)	530 (18.1%)
External causes of morbidity and mortality	320 (16.3%)	107 (22.9%)	37 (9.7%)	10 (9.0%)	474 (16.2%)
Diseases of the nervous system	325 (16.6%)	59 (12.6%)	31 (8.2%)	11 (9.9%)	426 (14.6%)
Certain infectious and parasitic diseases	90 (4.6%)	12 (2.6%)	14 (3.7%)	3 (2.7%)	119 (4.1%)
Endocrine, nutritional and metabolic diseases	36 (1.8%)	7 (1.5%)	58 (15.3%)	18 (16.2%)	119 (4.1%)
Mental and behavioural disorders	69 (3.5%)	16 (3.4%)	9 (2.4%)	1 (0.9%)	95 (3.3%)
Neoplasms (in situ, benign, uncertain or unknown behaviour)	64 (3.3%)	9 (1.9%)	9 (2.4%)	3 (2.7%)	85 (2.9%)
Diseases of the musculoskeletal system and connective tissue	57 (2.9%)	13 (2.8%)	7 (1.8%)	5 (4.5%)	82 (2.8%)
Diseases of the genitourinary system	37 (1.9%)	7 (1.5%)	21 (5.5%)	6 (5.4%)	71 (2.4%)
Symptoms, signs and abnormal clinical and laboratory findings, not elsewhere classified	43 (2.2%)	8 (1.7%)	10 (2.6%)	4 (3.6%)	65 (2.2%)
Diseases of the blood and blood-forming organs and certain disorders involving the immune mechanism	20 (1.0%)	2 (0.4%)	5 (1.3%)	1 (0.9%)	28 (1.0%)
Congenital malformations, deformations and chromosomal abnormalities	22 (1.1%)	4 (0.9%)	1 (0.3%)	0 (0.0%)	27 (0.9%)
Other death (temporary code)	12 (0.6%)	1 (0.2%)	0 (0.0%)	1 (0.9%)	14 (0.5%)
Diseases of the skin and subcutaneous tissue	7 (0.4%)	3 (0.6%)	1 (0.3%)	1 (0.9%)	12 (0.4%)

ESM Table 8: Measures of additive and multiplicative interaction between depression and diabetes on risk of all-cause and cause-specific mortality

	**Additive interaction**			**Multiplicative interaction**
	RERI (CI)	***AP (CI)***	***S (CI)***	***p-value***
**All-cause mortality**	0.29 (0.03 – 0.54)	0.13 (0.03 – 0.24)	1.32 (1.05 – 1.67)	0.182
**Cancer mortality**	0.38 (0.07 – 0.68)	0.23 (0.08 – 0.39)	2.57 (1.27 – 5.23)	0.006
**Circulatory mortality**	-0.03 (-0.52 – 0.46)	-0.01 (-0.24 – 0.21)	0.98 (0.65 – 1.46)	0.578
**Mortality from other causes**	0.26 (-0.48 – 1.01)	0.07 (-0.12 – 0.27)	1.11 (0.83 – 1.49)	0.061
**CVD mortality**	-0.26 (-0.85 – 0.33)	-0.11 (-0.39 – 0.17)	0.83 (0.54 – 1.29)	0.197
**Non-CVD circulatory mortality**	0.57 (-0.31 – 1.46)	0.29 (-0.06 – 0.63)	2.33 (0.68 – 7.98)	0.177

AP: Attributable proportion, RERI: Relative excess risk for interaction, S: Synergy index

ESM Table 9: HRs (95% CIs) for CVD- and non-CVD circulatory mortality risk among UK Biobank participants with neither, one of both of depression and diabetes

**Causes of mortality**	**Exposure**	**Unadjusted HR (95%CI)**	**Adjusted HR (95%CI)**	
			Model 1[a]	Model 2[b]
**CVD mortality**	Neither depression nor diabetes	1.0	1.0	1.0
	Depression alone	1.52 (1.32 – 1.76)	1.62 (1.39 – 1.88)	1.30 (1.12 – 1.52)
	Diabetes alone	5.82 (5.21 – 6.49)	3.27 (2.92 – 3.66)	2.25 (1.99 – 2.55)
	Depression and diabetes	6.96 (5.54 – 8.75)	4.20 (3.33 – 5.29)	2.29 (1.80 – 2.92)
**Non-CVD circulatory mortality**	Neither depression nor diabetes	1.0	1.0	1.0
	Depression alone	1.33 (1.04 – 1.71)	1.41 (1.09 – 1.81)	1.19 (0.92 – 1.54)
	Diabetes alone	3.07 (2.45 – 3.86)	1.88 (1.49 – 2.37)	1.24 (0.96 – 1.59)
	Depression and diabetes	5.89 (3.97 – 8.74)	3.92 (2.63 – 5.83)	2.00 (1.31 – 3.06)

a Model 1: adjusted for age, sex, ethnicity, education, income and area-based deprivation
b Model 2: adjusted as in model 1 plus for BMI, physical activity level, alcohol intake, smoking status, fruit and vegetable intake, oily fish intake, high cholesterol level, hypertension, history of CVD, history of cancer, family history of CVD and family history of depression at baseline

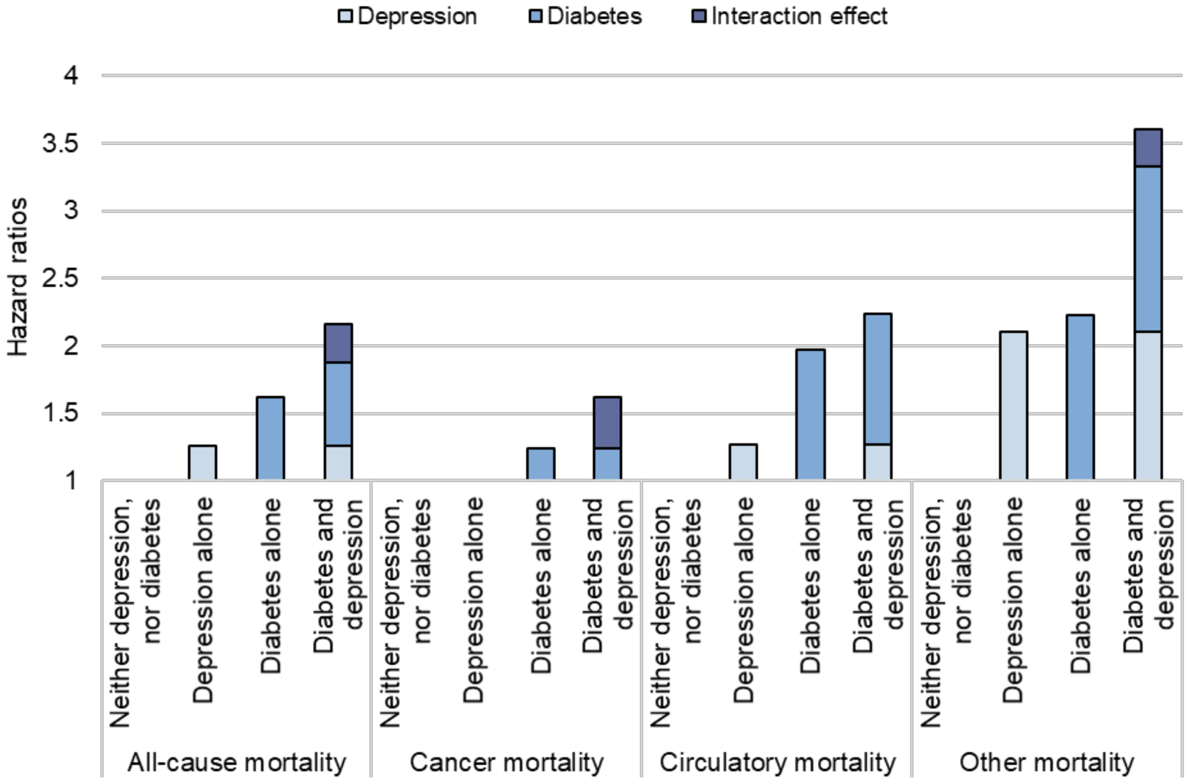

ESM Fig. 1: HRs (95% CIs) for all-cause and cause-specific mortality risk among UK Biobank participants with neither, one of both of depression and diabetes (fully adjusted models[a])

a Fully adjusted model: adjusted for age, sex, ethnicity, education, income and area-based deprivation, BMI, physical activity level, alcohol intake, smoking status, fruit and vegetable intake, oily fish intake, high cholesterol level, hypertension, history of CVD, history of cancer, family history of CVD and family history of depression at baseline

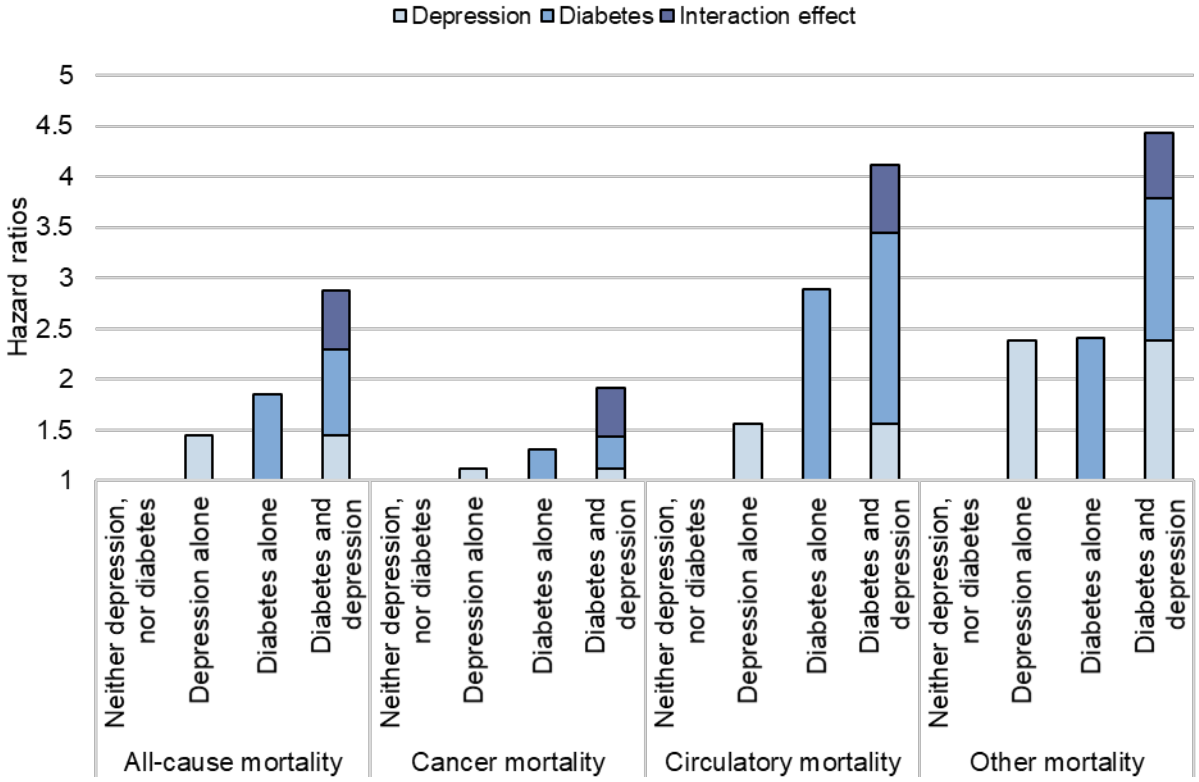

ESM Fig. 2: HRs (95% CI) for all-cause and cause-specific mortality among UK Biobank participants with neither, one of both of depression and diabetes (partially adjusted models*)

a Partially adjusted model: adjusted for age, sex, ethnicity, education, income and area-based deprivation
